# Sonographic chest B-lines anticipate elevated B-type natriuretic peptide level, irrespective of ejection fraction

**DOI:** 10.1186/s13613-015-0100-x

**Published:** 2015-12-30

**Authors:** Zouheir Bitar, Ossama Maadarani, Khaled Almerri

**Affiliations:** Department of Internal Medicine, Ahmadi Hospital, Kuwait Oil Company, Fahahil, Al Ahmadi, P.O. Box 46468, 64015 Kuwait city, Kuwait; Department of Cardiology, Chest Disease Hospital, Al Shuwaikh, Kuwait city, Kuwait

**Keywords:** Ultrasound chest, B-lines

## Abstract

**Background:**

Echocardiography and the *N*-terminal pro-brain-type natriuretic peptide (NT-proBNP) level are important tests for assessing left ventricular function in patients presenting to the emergency department with acute dyspnea. Chest ultrasound is becoming an important tool in diagnosing acute pulmonary edema.

**Aim:**

To assess the diagnostic accuracy of chest ultrasound examination using echocardiography and a curvilinear probe for detecting B-lines in patients presenting with acute pulmonary edema compared with assessment using NT-proBNP.

**Methods:**

This paper reports a prospective observational study of 61 consecutive patients presenting with symptoms and signs of pulmonary edema and B-profile detected by echocardiography with a 5 MHz curvilinear probe. The emergency department physicians ordered NT-proBNP levels, and critical care physicians trained in ultrasound examination performed echocardiography and chest ultrasounds. The findings of the chest ultrasound were reviewed by another senior physician.

**Results:**

Sixty-one participants were enrolled over a period of 6 months (49.2 % male, with a mean age 66.8). Forty-seven of the 61 patients had a B-profile. The median NT-proBNP level in the patients with B-profile was 6200, compared with the mean level in the patients with an A-profile of 180 (CI 0.33–0.82). The distributions in the two groups differed significantly (p = 0.034). Based on a threshold level of NT-proBNP in relation to age, the sensitivity and specificity (including the 95 % confidence interval) were determined; the sensitivity of finding B-profile on ultrasound was 92.0 %, and the specificity was 91.0 %. The positive predictive value of the B-profile was 97.0 %, and the negative predictive value was 71.0 %. The systolic function in the subjects with a B-profile was below 50 in 84.3 % of the subjects and normal in 15.7 % of the subjects. An A-profile was present in all of the subjects with systolic function >55 %. In the subjects with a B-profile, 94 % had a Framingham score of CHF >4; the subjects with all A-profile had scores <4, p < 0.0001. There was an NHANES score of >3 in 96 % of the subjects with a B-profile, and all of the subjects with an A-profile had scores <3 (p < 0.0001).

**Conclusions:**

Detecting the B-profile with an echocardiography probe (curvilinear 5 MHz) in lung ultrasound is highly sensitive and specific for elevated NT-proBNP helping in diagnosing pulmonary edema, although of resolution inferior to micro convex probes.

## Background

Acute pulmonary edema is a common problem facing emergency department (ED) physicians, and a percentage of these patients are admitted to a CCU. The diagnosis of acute pulmonary edema remains a challenge for the following reasons: the presentation could be in combination with other diseases, such as chronic obstructive airway disease; and other diseases have a presentation that is similar to that of acute pulmonary edema.

Cardiologists and intensivists commonly assess the heart using echocardiography. To save time, an extended evaluation could be performed using the same probe to complete the evaluation without changing the probe. Chest ultrasound is used to detect subpleural interstitial edema lines (B-lines) and pleural effusion; this method is used to exclude other important diseases, e.g., pneumothorax.

The assay for plasma ProBNP is a useful test for the evaluation of patients with dyspnea, and it is particularly useful as a component of the evaluation of a suspected heart failure when the diagnosis is uncertain [1].

The correlation between ProBNP and B-lines was studied using a linear probe. To simplify and expedite the procedure, we correlated the B-lines detected by the echocardiography probe to the NT-proBNP assay.

## Methods

### Study design

This work was a prospective, blinded observational study of an enrolled sample of ED patients in whom the ED physician requested NT-proBNP.

### Population and setting

The study was performed in the Ahmadi hospital ED and CCU. The hospital is a subsidiary of Kuwait Oil Company (KOC). The hospital serves KOC members and their families, including their parents, with annual ED census of 60,000 visits. US examinations are currently used in daily practice in the ED, CCU, and ICU of this hospital.

Patients were included if they were >18 years old and had acute dyspnea, and if the treating ED physician’s clinical suspicion that acute pulmonary edema and left ventricular failure was part of the differential diagnosis after the history and physical examination and before any testing was completed or a serum NT-proBNP was ordered in the ED. Clinical data were entered onto a separate standardized data collection form at the time of enrollment by the treating ED physician who was blinded to the ultrasound results. Clinical data included the patients’ age and sex, presenting symptoms, medical history, and physical examination findings, oxygen saturation from pulse oximetry and chest radiograph as listed in Table 1. For patients with a diagnosis other than left ventricular failure, confirmation was attempted using normal chest radiograph findings (absence of cardiomegaly or pulmonary venous congestion); radiographic signs pneumonia, or lung cancer. The ED physician were presented with the components and a summary of the Framingham scores for congestive heart failure (two major, or one major and two minor criteria), and the National Health and Nutrition and Examination Survey (NHANES) heart failure score (≥3) calculated from the case report form along with other information from the emergency department data sheets. We excluded patients if they were non-KOC members because the follow-up would be in other hospitals in Kuwait. Additionally, we excluded patients who were incapable of providing informed consent.

**Table 1:** The clinical characteristics of the patients in relation to ultrasound chest profiles

	A-profile no (%)	B-profile no (%)	Total no (%)	p
Medical history				
Myocardial infarction	0 (0.0)	37 (72.5)	60.7 (37)	<0.0001
Angina	3 (30.0)	27 (52.9)	30 (49.2)	0.164
Coronary artery bypass	0 (0.0)	12 (23.5)	12 (19.7)	0.091
Graft				
Atrial fibrillation	0 (0.0)	10 (19.6)	10 (16.4)	0.142
Hypertension	6 (60.0)	45 (88.2)	51 (83.6)	0.049
NIDDM	7 (70.0)	45 (88.2)	52 (85.2)	0.99
IDDM	3 (30.0)	6 (11.8)	9 (14.8)	0.157
Asthma	2 (20.0)	3 (5.9)	5 (8.2)	0.185
Obstructive airway disease	4 (40.0)	6 (11.8)	10 (16.4)	0.49
Chronic renal impairment	1 (10.0)	23 (45.1)	24 (39.3)	0.037
Clinical examination				
Shortness of breath	12 (91)	45 (95.7)	57 (93.4)	0.02
Elevated jugular venous pressure	2 (14)	33 (70)	35 (57)	<0.0001
Pulmonary rales	4 (28)	29 (61.7)	33 (54)	<0.0001
Wheezing	5 (30)	13 (28)	18 (29.5)	0.85
S3 gallop	2 (14)	35 (74.4)	37 (60.6)	<0.0001
Hypoxemia	12 (85.7)	43 (89)	55 (90)	0.02
Chest radiograph findings				
Normal heart size	11 (78.5)	11 (23.4)	22 (36)	<0.0001
Pulmonary venous congestion	2 (14)	43 (89)	45 (73.7)	<0.0001
Interstitial edema	1 (7)	40 (85)	41 (67)	<0.0001
Alveolar edema	1 (7)	40 (85)	41 (67)	<0.0001
Heart failure scores				
Framingham score^a^ > 2	0 (0)	47 (94)	47 (78.3)	<0.0001
NHANES scores^b^ > 3	0 (0)	48 (96)	48 (80)	<0.0001

^a^Two major (one point each) or one major and two minor (0.5 point each) criteria

^b^National health and nutrition examination survey

### Protocol

Each patient underwent an 8-zone thoracic ultrasound. The treating physician in the ED obtained informed written consent as well as baseline demographic and clinical data. Ultrasound scans were performed by a physician trained in the techniques of chest ultrasound and echocardiography. The ultrasound images were saved to a hard drive and reviewed in the system by a senior intensivist trained in critical care ultrasound. The physicians were blinded to the NT-proBNP results.

Ultrasound was performed using portable echocardiography (GE Vivid S6N, N-3191 Horten, Norway) equipment with a 5-MHz broadband curvilinear transducer in the echocardiography preset.

The exams consisted of bilateral scanning of the anterior and lateral chest wall and were performed with the patients in the supine or near-to-supine position. The correct scan was intercostal with the maximum extension of the visible pleural line. The chest wall was divided into eight areas, and scans for each area were obtained. The areas included two anterior and two lateral regions per side. The anterior chest wall was delineated from the sternum to the anterior axillary line and was subdivided into upper and lower halves (approximately from the clavicle to the second-third intercostal spaces and from the third intercostal space to the diaphragm). The lateral zone was delineated from the anterior to the posterior axillary line and was subdivided into upper and basal halves. The probe was placed in a cephalic orientation, and the pleural line was placed in the middle of the image by adjusting the depth settings [2].

### Measurements

**Fig. 1:**
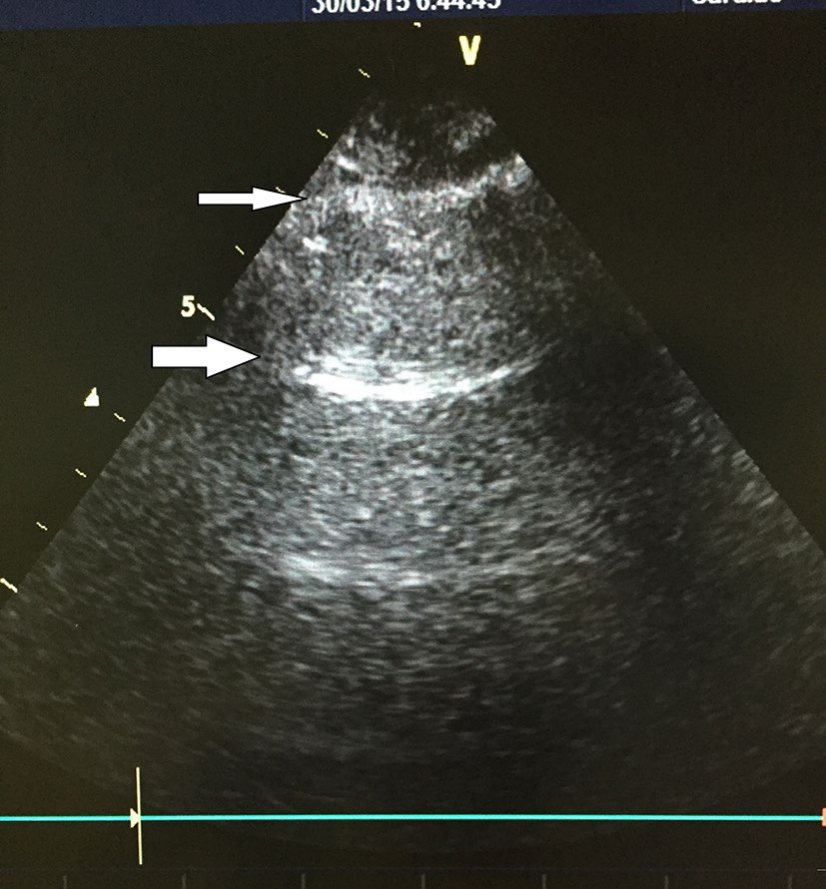
A-profile, pleural line (*small arrow*) visible between two ribs. Roughly horizontal parallel reverberation lines (*large arrows*). A-lines, shown in the figure, must be associated with lung sliding

**Fig. 2:**
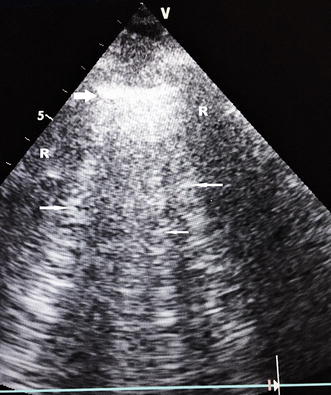
B-profile. Pleural line (*large arrows*) and here three B-lines (*small arrows*). Lung sliding, fully part of the definition of the B-profile, is not featuring here. *R* is acoustic rib shadow

The two primary findings on thoracic ultrasound are A-lines and B-lines. AB-line is a comet tail artifact that arises from the pleural line and moves in concert with lung sliding. It is long, well-defined, laser-like, and hyperechoic and it erases A-lines [2]. The updated definition of B-line requires three constant criteria (comet tail, arising from pleural line and moving with lung sliding) and four quite constant criteria (long, well-defined, hyperechoic, and erasing A-line) [3]. An A-line is the repetition of the pleural line, and it is an approximately horizontal hyperechoic line parallel to the pleural line [2]. Two important profiles are detected, as follows: the A-profile (Fig. 1) associates anterior lung sliding with A-lines, and the B-profile (Fig. 2) is defined by the presence of three or more B-lines in a longitudinal plane between two ribs per scan area, diffuse B-lines in more than one scan per side, and the presence of B-lines in both sides, associates anterior lung sliding [4].

### Data analysis

The statistical analyses were performed using SPSS 19. We determined the median Pro BNP value for the patients with A-profile and B-profile and calculated 95 % confidence intervals (CI) of the difference.

The evaluation of the data was performed with the Mann–Whitney U test to delineate the significance of the difference in the rank order of NT-proBNP in the patients with A-profile and B-profile.

The NT-proBNP level was considered positive at the threshold limit in relation to age. For age <50, the threshold limit for being positive was 450 pg/mL, for age 50–75, the level was 900, and for age >75, the level was 1800 pg/mL. A level below 300 excludes a diagnosis of heart failure with a negative predictive value of 80 % [5].

To increase the sensitivity of B-lines and chest ultrasound in cases of elevated NT-proBNP to reach a diagnosis in patients with dyspnea, we included the HANES criteria for the diagnosis of heart failure and the ejection systolic function (EF) when performing the echocardiography.

## Results

We enrolled 61 patients over a period of 6 months. The patients provided signed consent. The mean age was 66.8 years, with a range of 44–93 years; 49.2 % of the subjects were male. A total of forty-seven of the 61 patients had B-profile and hemodynamic pulmonary edema. The clinical characteristics of the patients in relation to ultrasound chest profiles are shown in Table 1.

The analysis revealed that A-profile was present in all of the patients with a NT-proBNP <400 or 300 pg/mL. Fifty of the 61 patients had elevated ProBNP levels in relation to age (Table 2).

**Table 2:** Chest ultrasound profiles based on *N*-terminal pro-brain-type natriuretic peptide

Thoracic ultrasound profile	**proBNP positive in relation to age**	**Negative proBNP**	**Total**
B-profile	46	1	47
A-profile	4	10	14
Total	50	11	61

Variable	**Value**	**95** **% confidence interval**
Sensitivity	0.92	0.812–0.968
Specificity	0.91	0.623–0.98
Positive predictive value	0.97	0.889–0.996
Negative predictive value	0.714	0.454–0.883
LR+	10.12	1.559–65.697
LR−	0.088	0.034–0.229

*LR+* positive likelihood ratio, *LR*− negative likelihood ratio

The median NT-proBNP levels in patients with B-profile was 6200, compared with a median of 180 in the subjects with A-profile (CI 0.33–0.82).

The distributions in the 2 groups differed significantly (Mann–Whitney U-static 3.98). Based on the threshold level of NT-proBNP of 250, the sensitivity of detecting B-profile on ultrasound was 95.0 %, and the specificity was 92.0 %. The positive predictive value of the B-profile was 90.0 %, and the negative predictive value was 88.0 % (Table 2).

The systolic function in the subjects with a B-profile was below 50 % in 84.3 % of cases and normal in 15.7 % of cases. The subjects with an A-profile all had systolic function >55 %.

Of those subjects with a B-profile, 94 % had a Framingham score of CHF >4; all of the subjects with an A-profile had a score of <4, p < 0.0001. National Health and Nutrition Examination Survey (NHANES) scores of >3 were found in 96 % of those with a B-profile, and all of the subjects with an A-profile had scores <3, p < 0.0001.

## Discussion

Lung ultrasound is becoming an essential tool for the diagnosis of pulmonary disease irrespective of the echocardiographic findings. This method is becoming a standard method to complement conventional Doppler echocardiography in the rapid evaluation of patients presenting with dyspnea in the emergency department (for the differential diagnosis of dyspnea), in hospital management (for serial evaluations in the same patient and for tailoring diuretic therapy), in the prehospital emergency setting (with hand-held echocardiography), and in the stress echocardiography lab (as a sign of acute pulmonary congestion during stress) [4].

Providing a reliable and repeatable estimation of EVLW, B-profile assessment by lung ultrasound represents a new, helpful tool for the cardiologist; this tool could be employed at all stages of the management of heart failure patients and could be used in the differential diagnosis of dyspnea [4, 6].

Increased left ventricular filling pressure is a common hemodynamic trigger for natriuretic peptides and B-profile. Wall distension is generally considered the main mechanical stimulus for natriuretic peptide production by ventricular tissue from stretched cardiomyocytes [7]. The use of NT-proBNP and B-type natriuretic peptide at the rule-out threshold recommended by the recent European Society of Cardiology guidelines on heart failure provides excellent ability to exclude acute heart failure in the acute setting with reassuringly high sensitivity [8]. The specificity of the natriuretic peptides is modest and variable, and therefore confirmatory diagnostic testing by cardiac imaging is required in the case of positive results [8].

The presence of B-profile as a sign of pulmonary interstitial edema is linked to augmented left ventricular filling pressures, which unbalance Starling forces at the alveolar–capillary barrier, resulting in pulmonary congestion [9]. Therefore, the overall good concordance and significant correlation between B-lines and NT-proBNP found in this study are not surprising.

The presence of B-profile correlating with higher BNP levels were previously studied using a linear probe because this method is easily learned and reproducible [10]. Although earlier literature identified similar results with curved probes [11], we used a curved probe in this study to allow for extended echocardiography examinations of the patient’s heart and chest using the same probe. The use of an echocardiography probe in detecting B-profile in the bedside evaluation of patients with known or suspected heart failure by experienced and inexperienced echocardiologists was previously studied and shown to be a reliable diagnostic tool [12]. Cardiac probes can show lung artifacts, may be with a quality inferior to some other equipment. We used an 8-zone scanning protocol compared with an NT-proBNP, and we detected that minimal pleural effusion corresponded more closely with heart failure in cases in which the patient presented with acute dyspnea. An eight-lung window protocol was used in the studies by Liteplo et al [11] and Volpicelli et al [2]. Lichtenstein et al. used a six-window protocol in previous studies and included the PosteroLateral Alveolar and/or Pleural Syndrome (PLAPS)-point, a posterior area accessible in supine patients, locating all free effusions, regardless of their volume [4].

In the literature, there have not been stringent criteria defining how many B-lines constitute a significant lung ultrasound finding. Lichtenstein et al. first used the presence of at least three B-lines per field of scan seen longitudinally between two ribs, with a distance between two B-lines <7 mm, as criteria for abnormality, and the majority of authors have followed this convention (1, 2, 10, 13). These criteria have been used irrespective of the scan technique, whether the scan is transverse in the intercostal space or longitudinal across ribs. Typically, a micro convex or phased array probe with a narrow footprint is used. Some operators decide to use a linear or curvilinear probe with a broad footprint. These probes allow for views across several rib spaces or for a longer view of the pleural line. A positive scan with these probes should be similar to the general convention of at least three B-lines less than 7 mm apart [14].

The presence of B-profile is specific for an elevated NT-proBNP level although the sensitivity is variable [15]. In a linear probe study, the sensitivity was 33.3 %. B-lines had a sensitivity of 100 % and a specificity of 92 % in the diagnosis of pulmonary edema when compared with COPD [16]. Gargani L et al. used a cardiac probe and determined that B-lines are reliable in predicting the cardiogenic origin of dyspnea, with an accuracy comparable to natriuretic peptides [13].

The echocardiographic data appear useful in the context of a lung ultrasound examination, given the high accuracy published in previous articles (Sensitivity 97 %, specificity 95 %) [3]. B-profile could be a plausible alternative in acute settings where natriuretic peptide analysis is not available or when there is insufficient time to perform the assay, as occurs in patients with rapidly developing acute respiratory failure. The estimated time in getting the result of NT-proBNP after sampling of the patient is almost 3 h in our institute.

The presence of B-profile correlated well with the clinical scores of heart failure in our study. A total of 94 % of the patients with a B-profile had a Framingham score of CHF >4, and 96 % had NHANES scores of >3. All of the patients with A-profile had normal scores. Our study demonstrates the clinical correlation of chest ultrasound examinations with heart failure scores, as showed previously in the BLUE-protocol study [3].

Chest sonography focused on B-profile when combined with a comprehensive echocardiography study could evaluate systolic and diastolic cardiac function [15]. Patients with cardiogenic edema are more likely to have a lower ejection fraction and a higher degree of diastolic dysfunction [17]. In our study, 15.4 % of patients with cardiogenic pulmonary edema and B-profile had a left ventricular ejection fraction (LVEF) >50 % with high NT-proBNP levels, predominantly because of diastolic dysfunction.

Limitations of the study include low number of patients recruited. This was due to two main reasons; NT-proBNP was ordered by the ED physician on difficult patients with high heart failure scores, thus missing patients with genuine hemodynamic pulmonary edema. Thus, other profiles of BLUE-protocol in our population were not included. Furthermore, patients incapable of providing informed consent, even though having pulmonary edema, were omitted from the study. We compared the B-profile to the clinical signs of heart failure and to a correlation with natriuretic peptides. Spectral tissue Doppler-derived index(E/E’) for assessing left atrial pressure as well as patients having heart failure with preserved ejection fraction were not studied, thus considered a limitation to our study.

## Conclusion

Detecting the B-profile is highly sensitive and specific for elevated NT-proBNP helping in diagnosing pulmonary edema. A cardiac probe (curvilinear 5 MHz) can be used, although of resolution inferior to some micro convex probes. Performing chest ultrasound could be part of the echocardiography evaluation in patients with acute dyspnea.
